# Global Systematic Review of the Cost-Effectiveness of Indigenous Health Interventions

**DOI:** 10.1371/journal.pone.0111249

**Published:** 2014-11-05

**Authors:** Blake J. Angell, Janani Muhunthan, Michelle Irving, Sandra Eades, Stephen Jan

**Affiliations:** 1 The Poche Centre for Indigenous Health and the George Institute for Global Health, The University of Sydney, Sydney, New South Wales, Australia; 2 The George Institute for Global Health, The University of Sydney, Sydney, New South Wales, Australia; 3 The Poche Centre for Indigenous Health, The University of Sydney, Sydney, New South Wales, Australia; 4 Baker IDI, Sydney, New South Wales, Australia; Quensland University of Technology, Australia

## Abstract

**Background:**

Indigenous populations around the world have consistently been shown to bear a greater burden of disease, death and disability than their non-Indigenous counterparts. Despite this, little is known about what constitutes cost-effective interventions in these groups. The objective of this paper was to assess the global cost-effectiveness literature in Indigenous health to identify characteristics of successful and unsuccessful interventions and highlight areas for further research.

**Methods and Findings:**

A systematic review of the published literature was carried out. MEDLINE, PSYCINFO, ECONLIT, EMBASE and CINAHL were searched with terms to identify cost-effectiveness evaluations of interventions in Indigenous populations around the world. The WHO definition was followed in identifying Indigenous populations. 19 studies reporting on 27 interventions were included in the review. The majority of studies came from high-income nations with only two studies of interventions in low and middle-income nations. 22 of the 27 interventions included in the analysis were found to be cost-effective or cost-saving by the respective studies. There were only two studies that focused on Indigenous communities in urban areas, neither of which was found to be cost-effective. There was little attention paid to Indigenous conceptions of health in included studies. Of the 27 included studies, 23 were interventions that specifically targeted Indigenous populations. Outreach programs were shown to be consistently cost-effective.

**Conclusion:**

The comprehensive review found only a small number of studies examining the cost-effectiveness of interventions into Indigenous communities around the world. Given the persistent disparities in health outcomes faced by these populations and commitments from governments around the world to improving these outcomes, it is an area where the health economics and public health fields can play an important role in improving the health of millions of people.

## Introduction

There are almost 400 million Indigenous people living in countries around the world [1]. The World Health Organisation (WHO) defines Indigenous populations as those that live in distinct geographical territories, identify themselves as belonging to a cultural group separate from mainstream society and are descendent from groups present in the area before modern states and borders were defined [2]. Indigenous populations have been repeatedly shown to bear a greater burden of death, disease and disability than their non-Indigenous counterparts [1], [3]–[6]. Despite this, little is known about what constitutes cost-effective health interventions in these unique population groups and there is a lack of evidence as to the extent and nature of investment in programs to address the burden of ill-health in these populations [7],[8].

Economic evaluation of health care programs has become an important area of applied economics over the last 30 years [8]–[10]. The field has had a significant impact on policy-making processes in countries around the world most prominently through high level policy initiatives such as the National Institute for Health and Care Excellence in the UK and the Pharmaceutical Benefits Advisory Committee in Australia. Economic evaluation analyses whether the additional benefits of an intervention is worth undertaking relative to another intervention or normal care [9]. Without a substantial evidence-base on the cost-effectiveness of policy options, policy-makers have little economic evidence to make resource allocation decisions in the field of Indigenous health.

The objective of this review is to systematically search the literature to pull together existing evaluations that estimate the cost-effectiveness of health interventions into Indigenous populations around the world. This review allows for an exploration of the type of interventions that have been shown to be effective in these unique population groups, the specific resource requirements needed to deliver programs to these populations and the aspects of these programs that are deemed to be of value by the populations to whom they are targeted.

### Suitability of the traditional health economic paradigm for the Indigenous health field

There is an increasing recognition of the shortcomings of traditional methods of measuring health benefits in economic evaluations of healthcare programs [8],[10]–[12]. Indigenous populations have been shown to hold different conceptions of health to mainstream populations [1],[8]. Specifically, family, community, connections to the land and cultural sensitivity have been shown to be qualitatively valued with regards to health by Indigenous populations [8]. While this has led some to argue that the traditional approaches of the health economic paradigm are inappropriate to measure the impact of interventions in these communities, it highlights the importance of further economic research into the field and suggests that valuation of outcomes within economic studies should ideally incorporate some form of patient or user-elicited valuation so that they reflect these broader conceptions of health.

To provide a basis for further evaluating these arguments, this review also highlights any explicit attempts by included studies to incorporate these concerns into cost-effectiveness evaluations.

## Methods

A systematic review of the literature was conducted to find articles that provide an economic evaluation of interventions targeting or reporting on an Indigenous population. No protocol has been previously published for this review.

### Inclusion Criteria

The inclusion criteria for this review specified three characteristics for studies. First, the studies had to examine interventions that were primarily aimed at improving the health of target populations. Second, included papers had to be economic-evaluations of an intervention that met the definition of one of the types outlined in Table 1. This depicts a broad spectrum of economic evaluation methodologies from cost-benefit analysis, generally considered the form of economic evaluation that is most comprehensive in scope, to the narrower forms of evaluation including simple cost-analyses. The search strategy was adapted from previously published systematic reviews of economic evaluations [13],[14]. Studies were required to report on either the cost impacts of the intervention of interest or some measure of cost-benefit to be included in the study. Finally the papers had to either focus on or separately report on a population that is either wholly or partially Indigenous. The WHO definition of Indigenous mentioned above was used as a basis for identifying these populations in the literature [2]. The specific search strategy was adapted from a previously published systematic review of this population group and is designed to encompass Indigenous populations around the world in line with this definition [15].

**Table 1:** Types of Economic Evaluation.

Cost Analysis	A partial form of economic analysis where only the costs of an intervention are compared to another potential intervention.
Cost-Effectiveness Analysis	Provide a measure of the effectiveness of an intervention using natural units such as life-years gained relative to the cost incurred to obtain that outcome.
Cost-Utility Analysis	A particular form of Cost-Effectiveness Analysis that measures effectiveness using a measure of utility such as Quality Adjusted Life Years or Disability Adjusted Life Years.
Cost-Benefit Analysis	An economic evaluation that sees both the costs and benefits of a particular intervention valued in monetary terms.

### Search strategy

A search was conducted of CINAHL, MEDLINE, EMBASE, PSYCINFO and ECONLIT (from inception to May 2014) using variations of the search string contained in Table 2. Reference lists of included studies were also searched for further applicable studies.

**Table 2:** Search Strategy.

Database/s	Search terms
Cinahl, Medline, EMBASE and PsycINFO	(1) The following terms as words within the title, abstracts or texts of papers: aborigin* or american indian* or eskimo* or Ethnic Group* or first nation* or greenlandic or indigenous or inuit* or inupiat* or inuvialuit* or kalaallit* or maori or maoris or mapuche* or native american* or native people* or native population* or native siberian* or navaho* or nunangat* or sami* or skolt* or taiga* or tribe or tribal or yuit or yupik or zuni(2) *“cost-effectiveness” or “economic evaluation” or “cost impact”* as words within the title, abstracts or texts of papers or containing a subject heading under “*cost analysis/”*
Econlit	(3) Econlit was also searched with *“Health”* as a subject

### Data Extraction

Study review, selection and data extraction were independently undertaken by two authors (BA and JM). Abstracts, titles and keywords of the studies returned from the search were screened for compatibility with the inclusion criteria. Once studies were identified for potential inclusion, full texts were reviewed. Data were extracted from the studies using a form developed for the review based on standard techniques used in the literature and included the following items: country of origin, methodology including type of evaluation, comparators used, outcome measures, settings and participants, results and evidence of inclusion of Indigenous conceptions of health [16]. The primary outcome measures were reported measures of cost-effectiveness.

## Results

**Figure 1:**
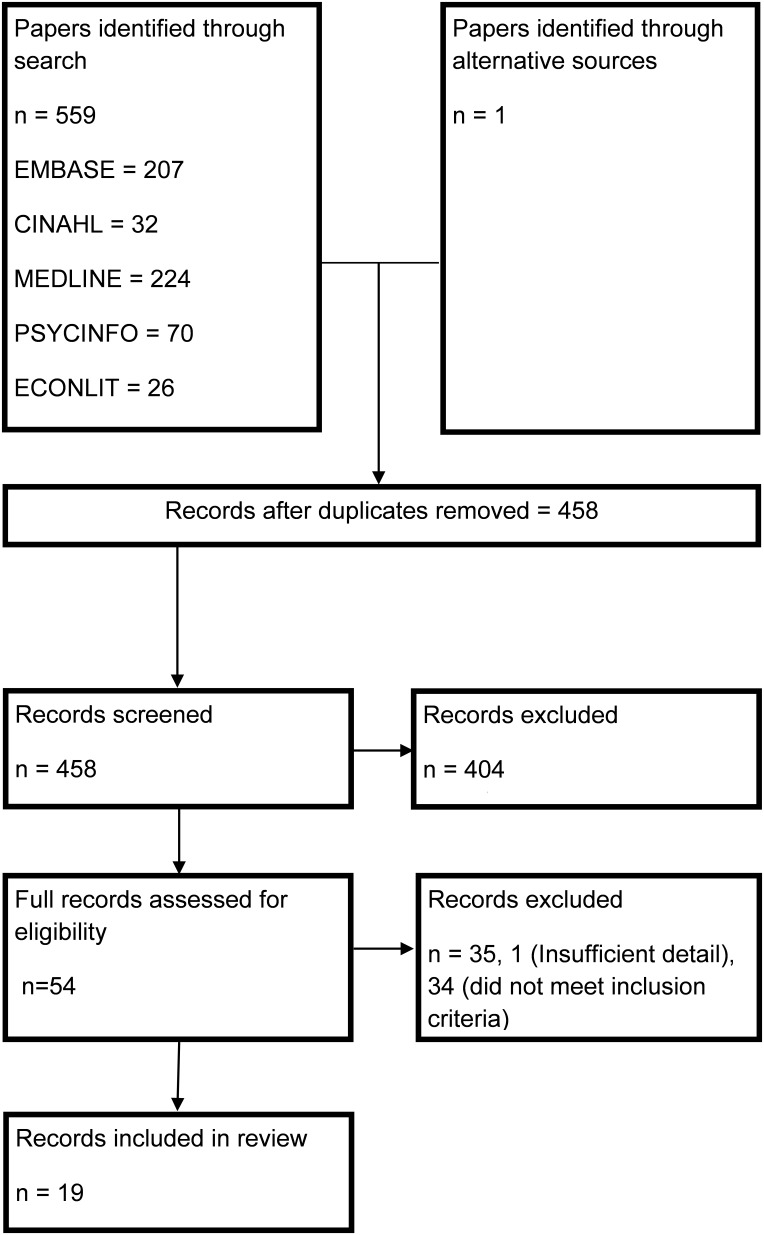
PRISMA Flowchart.

The search yielded 559 abstracts (see Figure 1). One further study was identified through a hand search of relevant journals. Once duplicates were removed, 458 unique abstracts were reviewed. Three published abstracts were found that appeared to meet the inclusion criteria. Relevant authors were contacted to identify if further publications resulted from these abstracts. The authors of one study did not respond [17], one was published as a short-report that included no additional information to the abstract [18] while one was being readied for submission and not yet available to other researchers [19]. Two of the abstracts were included as studies as they contained enough information to meet the data extraction requirements [17],[18] while the other was excluded as there was insufficient information in the abstract to be included [19]. In total, nineteen studies reporting on 27 interventions met the criteria to be included in this review [7],[11],[17],[18],[20]–[34]. Tables 3 and 4 summarise the characteristics of included studies.

**Table 3:** Primary intervention Studies.

Study	Country	Setting	Intervention	Population	Comparator	Type of economic evaluation	Finding
McDermottandSegal(2006)	Australia	Remote primarycare centresand hospitals	Primary health care –visiting specialist serviceon top of improvedtraining for local staff,preparation of registers,care plans and recallsystems for patients withdiabetes and aninformation system thatcan report on care quality.	RemoteIndigenousIslanderpopulationwith diabetes	**Business as usual** – increases based on historicaltrends	Cost-analysis	**Cost-saving –**Net Present Valuecost saving witha 5% discountrate over 9 years.
Dyson,Kruger andTennant(2012)	Australia	Rural andremote dentalhealthservices	Dental health care(outreach) One-weekclinics of a visiting dentistassisted by an AboriginalHealth Worker. Transportwas provided as neededby local health servicesand all services providedwere free of chargeto the patient	Remotecommunitiespresenting withdental problems	Cost-Benefit ratioscompared to unpublished estimates of WAgovernment services aswell as weightingspublished byNew South Wales.	Cost-effectiveness	**Cost-Effective –** Costbenefit ratiosranged frombetween 1.22 and2.05 for different clinics.
Jin,Martinet al.(2004)	Canada	Remotecommunities,mobile clinic	Primary health care(outreach): Outreachservices comprising of adiabetes nurse educatorand an ophthalmictechnician offeringrecommended serviceswithin Aboriginal reserves	Diabeticresidents ofremote areas	Costs associatedwith accessingsimilar servicesin the absenceof the mobile clinic	Cost-analysis	**Cost-Saving –** Over the firstyear of operation the mean costper client was found to be$1231 (CDN) VS $1437 ifthese services had beenobtained elsewhere. Qualitativefindings showed the service tobe highly valuedby the local population.
Zaloshnjaet al.(2003)	USA	Rural andremote NativeAmericanReserves	Prevention regulatory;Enforcement of lawrequiring automobiledrivers and passengers towear safety beltsincluding promotionof the law andbenefits of safety belt use.	Remote Indigenous residents	Business as usual rates of injuries based on historical trends	Cost Benefit Analysis	**Cost-Effective –** benefit cost ratio (BCR) = 256
Zaloshnjaet al.(2003)	USA	Rural andremote NativeAmerican Reserves	Prevention (builtenvironment): Installationof streetlights along a darksection of highwaydesigned to reducepedestrian injuries.	Remote Indigenous residents	Business as usual rates of injuries based on historical trends	Cost Benefit Analysis	**Cost-Effective –** BCR = 10
Zaloshnja et al. (2003)	USA	Rural and remote Native American Reserves	Prevention (regulatory):Authority given toimpound free-roaminglivestock on reservation roads.	RemoteIndigenousresidents	Business as usual ratesof injuries based onhistorical trends	Cost BenefitAnalysis	**Cost-Effective –** BCR = 1.67
Zaloshnjaet al.(2003)	USA	Rural andremote NativeAmericanReserves	Prevention: Swimming andwater survival courses aswell as summer-weightcoats that served asecondary function asfloatation devices.	RemoteIndigenousresidents	Business as usualrates of injuriesbased onhistorical trends	Cost BenefitAnalysis	**Cost-Effective –** BCR = 2592
Zaloshnjaet al.(2003)	USA	Rural andremote NativeAmericanReserves	Prevention: Employment ofa social worker who spent80% of her time on suicideprevention supplementedby a school-based programwhich trained youthleaders to recognise warning signs of suicidal ideation.	RemoteIndigenousresidents	Business as usualrates of injuriesbased onhistorical trends	Cost BenefitAnalysis	**Cost-Effective –** BCR = 43
Wilsonet al.(2010)	USA	RegionalPrimaryMedicalcentres	Primary health care(prescribing): Aggressivetargets of LDL-C andsystolic blood pressureversus standard targets.	American Indiansover the ageof 40 with typetwo diabetes andno prior cardiovascular events	Control groupreceiving usual care	Cost-Utility	**Cost-Ineffective –** Cost per QALY = $82,589
Shoreet al.(2007)	USA	Remote nativeAmericancommunities	Telehealth - psychiatricinterviews for American Indian Veterans conductedusing telehealth,digital networkequipment.	American IndianVeterans livingin remotecommunities	Non-intervention sitesreceiving in personinterviews (usual care)	Cost Analysis	**Cost-Effective –** Telehealth cost $6,000 more in 2003 but $8,000 less in 2005 for clinics that had to be set up. For clinics already set up telehealth interviews in 2003 telehealth cost $1,700 more but in 2005 they cost $12,000 less.
Bakeret al.(2005)	Australia	RemotePrimary Care	Primary health care(prescribing): Perindoprilalong with other medicationas necessary and education	Aboriginal adultswith hypertension,diabetes withmicroalbuminuria or overtalbuminuria andovert albuminurialiving in remotecommunities	Business as usualincreases in costs andcases based onhistorical trends	Cost analysis	**Cost-saving -** $1 M net saving after 3 years and $3.4 M at 4.6 years
Janet al.(2004)	Australia	Urbanprimary care	Primary health care(midwifery): Aboriginalcommunity controlledmidwifery service	Aboriginalpregnantwomen	Aboriginal womennot in the programreceiving usual care	Cost analysis	**Not Cost-Saving -** Net cost to health sector estimated to be $1200 per client over normal care. Qualitative evaluation found service to be highly valued by recipients.
Fergussonet al. (2011)	Thailand	Remoteprimary care	Primary health care(screening): Serologicscreening prior to hepatitis Bvaccines in remote tribes	Akha tribalchildren inNorthernThailand	Usual care where allchildren receive thevaccine	Cost analysis	**Cost-Saving –** over two years $1104 and $1556 (USD) were avoided as a result of screening
Martin andYidegiligne(1998)	Canada	RemotePrimary Care	Primary health care(screening): Travelling retinalphotography screeningteam to see remote patients	AboriginalCanadians livingon remotereserves	Costs associated withaccessing similar servicesin the absence of themobile clinic	Cost Analysis	**Cost-Saving –** seeing diabetic patients with the traveling team would cost $103,000 (Canadian) less than if these patients had to travel to obtain the services
Gaoet al.(2014)	Australia	RemotePrimary Care	Primary health care(midwifery): Midwiferygroup practice where a groupof midwives offercontinuity of carethroughout the preand post pregnancy period.	RegionalAboriginalpregnant women.	Business as usualincreases in costs,presentations andcomplications basedon historical trends	Cost analysis	**Non-significant cost-savings** of $703 found to result from the program
Carvalhoet al.(2011)	Brazil	RemotePrimary Care	Primary health carescreening and treatment):Universal rapid syphilistesting and treatment.	Remote Amazonianpopulation –pregnant womenand the sexuallyactive population.	Argue that interventionis cost-effective relativeto costs associated withaccessing similar servicesin the absence of themobile clinic	Cost-UtilityAnalysis	**Cost-Effective –** Cost per DALY saved $484 (USD)

**Table 4:** Modelled Intervention Studies.

Study	Country	Setting	Intervention	Population	Comparator	Type of economic evaluation	Finding
Sheerin(2004)	New Zealand	Primary Care	Primary health care(vaccination?) Hepatitis Cvirus anti-viral therapy forinjecting drug users onmethadone maintenancetherapy as opposedto MMT on its own.	Modelled cohort of1000 injecting drugusers including Maoris.	No treatment	**Cost Effectiveness –**Markov model wasused with a lifetimefollow up to modelcost-effectiveness.	**Cost-Effective –** cost per life-year saved for Maori men ranged between $8,000 and $35,000NZD while for Maori women these numbers were $7,000-$23,000
Maberlyet al. (2003)	Canada	Remoteprimary care	Primary health care(outreach, screening)Retinopathy screening bytravelling retina specialistsversus retinal photographywith a portabledigital camera	Isolated First NationsCohort with diabetes	Usual care wherea specialist visitsthe regionalcentre and remotepatients are flownin for assessment.	**Cost Utility Analysis** –Markov models wereused to model theimplementation ofservices over 5 yearswith a total timeframeof 10 years for the analysis.	**Cost-Effective –** Portable retinal camera ($15,000 per QALY gained) found to be a more cost-effective means of screening for diabetic retinopathy than a retina specialist ($37,000 per QALY gained)
Onget al.(2012)	Australia	Primary care	1. Broad community basedintervention involving:regular health promotionactivities, physical activitygroups, smoking restrictionsin public buildings andimproved nutritional valueof food at local store.	Indigenous populationof Australia aged 35and above	Interventions weremodelled againsta situation ofusual care	**Cost Utility Analysis –**a decision analyticMarkov model wasused with a lifetimefollow-up (or untilindividuals reached85 years).	**Cost-Ineffective –** the intervention is modelled to cost$210,000AUD per DALY which was deemed cost-ineffective using a $50,000 cost per DALY threshold.
Onget al.(2012)	Australia	Primary care	2. Statins	Indigenous populationof Australia aged 35and above	Interventions weremodelled againsta situation ofusual care	**Cost Utility Analysis –**a decision analyticMarkov model wasused with a lifetimefollow-up (or untilindividuals reached 85 years).	**Cost-Ineffective –** $80,000AUD per DALY when delivered through Aboriginal Controlled Community Health Services (ACCHSs) and $59,000 when delivered through mainstream GPs
Onget al.(2012)	Australia	Primary care	3. Low dose diuretics	Indigenous populationof Australia aged 35and above	Interventions weremodelled againsta situation ofusual care	**Cost Utility Analysis –**a decision analyticMarkov model wasused with a lifetimefollow-up (or untilindividuals reached 85 years).	**Cost-Effective -** $31,000AUD per DALY through ACCHSs or $11,000AUD through mainstream GPs
Onget al.(2012)	Australia	Primary care	4. ACE inhibitors	Indigenous populationof Australia aged 35and above	Interventions weremodelled againsta situation ofusual care	**Cost Utility Analysis –**a decision analyticMarkov model wasused with a lifetimefollow-up (or untilindividuals reached 85 years).	**Cost-Effective –** $50,000AUD per DALY when delivered through ACCHSs and $31,000AUD through mainstream GPs
Onget al.(2012)	Australia	Primary care	5. Polypill	Indigenous populationof Australia aged 35and above	Interventions weremodelled againsta situation ofusual care	**Cost Utility Analysis –**a decision analyticMarkov model wasused with a lifetimefollow-up (or untilindividuals reached 85 years).	**Cost-Effective -** $21,000AUD per DALY when delivered from ACCHSs ($13,000AUD from mainstream GPs)
Panattoniet al.(2012)	New Zealand	Hospital	Treating all Acute CoronarySyndromes patients acrossNew Zealand with genericclopidogrel and usinggenetic testing	Entire ACS populationin New Zealandpublic hospitals	Non-geneticallyguided treatment	**Cost-Utility Analysis –**used a decision treemodel to projectclinical effectivenessdata over a lifetimefollow up.	**Cost-Effective -** The genetically guided strategy was particularly cost effective for Maoris ($NZ7312/QALY)
Reeve(2006)	Australia	Hospital	Palivizumabimmunoprophylaxis forinfants at risk	‘High-risk’ infantsincluding those withlow-birth weight andmothers who weremultiparous babies bornin an urban hospital.	Actual treatmentcosts (cases wereretrospectivelyidentified for theanalysis)	**Cost-effectiveness** **Analysis –** calculatedthe costs and projectedoutcomes if thesegroups of infants hadbeen treated rather thanreceiving actual care.	**Cost-Ineffective –** cost (only drug costs) per prevented admission ranged from $69,861 to $88,547AUD.
Rush(2014)	New Zealand	Schools	A nutrition and physicalactivity program designed tohelp reduce excess weightgain and risk ofchronic disease	All New Zealand schoolstudents up until grade 8.	Other studentsnot participatingin program	**Cost-Utility Analysis –**applied a previouslyused Markov modelwith a lifetime follow-up to determineeffectiveness	**Cost-Effective –** Cost per QALY gained in Maori population was given by - $28,241 for the younger group and older $22,151
Tamet al.(2009)	Canada	Hospital	Palivizumabinjections forinfants (<1 year)	Indigenous Inuitcommunities in either aregional centre orremote area.	No prophylaxisor usual care.	**Cost-Utility Analysis –**used a decisionanalytical model withlifetime follow-up usedin the analysis	**Cost-Effective -** For all infants the ICER was $39,435/QALY. Looking only at rural areas there was an associated ICER of $24,750/QALY. Prophylaxis was a dominant strategy (cost saving) for rural infants under 6 months of age.

### Country of Origin

Seven studies were conducted in Australia (covering eleven interventions) [7],[11],[20],[21],[25],[27],[33], four in Canada [22]–[24],[31] and three each in New Zealand [26],[28],[29] and the USA (covering seven interventions) [30],[32],[34]. A published abstract was included from both Thailand [17] and Brazil [18].

### Settings and Participants

Two studies evaluated interventions in urban areas, one looking at a midwifery program [11] and the other at palivizumab treatment for children [27]. Twelve studies focused solely on rural or remote locations [17],[18],[20]–[25],[30]–[34] while the remaining five studies were based on wider populations encompassing rural, remote and urban Indigenous communities [7],[26],[28],[29],[31].

The majority of the studies focused on Indigenous populations with known health conditions including diabetes (six studies all conducted in rural or remote locations) [20],[22]–[25],[32], post-traumatic stress disorder [30], heart conditions [26], dental problems [21], drug addiction [29] and pregnancy [11],[33]. The other intervention studies targeted population groups that were not based on the presence of some particular medical condition [7],[17],[18],[27],[28],[31],[34].

Of the 27 interventions included in the study, two were carried out in infants [27],[31], one targeted school children [28], another in children more generally [17], one targeted young adults [34], two specified older patients [26],[32] while the rest were not targeted at any specific age-groups [7],[11],[18],[20]–[25],[29],[30],[33],[34].

### Methodology

Studies were broadly grouped into two groups. The first included group included studies where authors collected effectiveness data within the study itself (twelve studies and sixteen interventions) [11],[17],[18],[20]–[22],[24],[25],[30],[32]–[34]. The second group included studies that used previous findings in the literature to model the impact of potential interventions on these populations (seven studies looking at eleven interventions) [7],[23],[26]–[29],[31].

The types of economic evaluation are outlined in Table 1. Only one study met the criteria of a cost-benefit analysis, generally considered the most comprehensive form of health economic evaluation [34]. Eight studies met the criteria of cost-analyses, the least complex of the four categories [11],[17],[20],[22],[24],[25],[30],[33]. There were three cost-effectiveness analyses [21],[27],[29] and seven cost-utility analyses [7],[18],[23],[28],[29],[31],[32].

### Outcome Measures

Costs were the primary outcome measure reported in eight of the studies [11],[17],[20],[22],[24],[25],[30],[33]. One study reported cost-benefit ratios for five injury-prevention interventions [34]. The study of a remote dental service in Western Australia attempted to use published valuations of equivalent government services as an estimate for the value of services provided and reported on the cost-benefit ratio in this regard [21]. Five studies reported costs per quality adjusted life years gained [23],[26],[31],[32] and two reported on cost per disability life years gained [7],[18]. Costs per life years saved were reported by Sheerin et al. in their study on Hepatitis C treatment for injecting drug users in New Zealand [29], while cost per avoided hospitalisation was the focus of the study of Reeve et al. on palivizumab injections for high risk infants [27].

Each included paper made some judgment as to the cost-effectiveness or cost-impact of the interventions being studied. In total, only five of the 27 interventions were deemed to be strictly not cost-effective or cost-saving [7],[11],[27],[32]. Of the eight cost-analysis studies, six found interventions would be cost-saving over time relative to the comparison [17],[20],[22],[24],[25],[30]. The study of the group midwifery program in the Top End of Australia found no significant cost differences between the provided intervention and usual care [33], while the urban midwifery program was deemed not cost-saving although qualitative results demonstrated that patients valued the service [11]. The cost-benefit analyses carried out all found the injury prevention interventions to be cost-beneficial in that they had benefit-cost ratios above one [34]. Two of the three cost-effectiveness studies found their interventions to be cost-effective [21],[29]. Three interventions analysed using cost-utility analysis were found to cost-ineffective [7],[32]. Table 5 depicts included interventions by cost-effectiveness.

**Table 5:** Included Interventions by cost-effectiveness.

*Cost-Effective Interventions*
Palivizumab in Indigenous infants
Multicomponent physical activity and nutrition program
Genetic testing for CYP2C19 Variants to guide thienopyridine treatment
Low dose diuretics for the prevention of cardiovascular disease
ACE Inhibitors for the prevention of cardiovascular disease
Polypill for the prevention of cardiovascular disease
Screening for diabetic retinopathy
Rapid syphilis testing
Hepatitis C treatment for injecting drug users on methadone maintenance programs
Safety-belt program
Installation of streetlights on remote highways to prevent crashes
Livestock control measures to prevent crashes
Drowning prevention program
Suicide prevention program
Outreach dental services
***Cost-Saving Interventions***
Screening for diabetic retinopathy
Screening for hepatitis B prior to vaccination
Perindopril for diabetes patients along with other medication as necessary and education
Telehealth for psychiatric interviews
Outreach diabetes services
Better training of local diabetes staff and visiting specialist
***Interventions with Non-Significant Cost Savings***
Midwifery group practice
***Cost-Ineffective Interventions***
Palivizumab for high-risk infants
Broadbased healthy living program to prevent cardiovascular disease
Statins to prevent cardiovascular disease
Lower targets for blood pressure and LDL cholesterol in diabetics
***Interventions that were not cost-saving***
Community-based midwifery service

### Comparators Used

The choice of comparator against which the cost-effectiveness of the intervention is assessed plays a large part in determining whether a particular intervention is cost-effective or not. Included studies could be grouped into two main categories in this regard. Fourteen of the studies assessed the cost-effectiveness of their intervention against so-called ‘business as usual’ cases where they were compared to a situation with no intervention, either through the use of a control group [11],[28],[30],[32], projecting historical trends [7],[17],[20],[25],[26],[29],[31],[33],[34] or in one case actual hospital records with the impact of the intervention being retrospectively assessed [27]. The remaining five studies compared the cost-effectiveness of the intervention to a hypothetical or alternatively modelled scenario where participants would be forced to obtain the intervention through alternative service providers [18],[21]–[24].

### Wider conceptions of health

Only one study that met the inclusion criteria explicitly set out to capture wider benefits of culturally appropriate service provision [11]. This was done through broader qualitative evaluation of the value of the provided midwifery service and the use of a cost-consequence approach designed to incorporate broader values than narrowly defined health outcomes. Another included study also included qualitative results from patient interviews to document the appropriateness of the service to the Indigenous community [22]. The work of the ACE prevention project in Australia modelled the different impact of delivering interventions via Aboriginal Medical Services and mainstream general-practitioners, suggesting that health benefits to Indigenous communities would be higher from those services delivered through Aboriginal Medical Services as a result of increased engagement of the target population [7]. The remaining studies did not explicitly attempt to measure any wider or Indigenous-specific conceptions of health. It is important to note, though, that of the 27 interventions included in the analyses, only four were not targeted specifically at Indigenous populations [26]–[29]. Of the modelled studies, six of the eleven interventions examined were based on estimates of intervention effectiveness that had been obtained from studies carried out in Indigenous populations [7],[26]–[29],[31]. The other five were based on effectiveness estimates emanating from the general non-Indigenous literature and applied to Indigenous populations.

## Discussion

This systematic review has found that very few cost-effectiveness studies are available in the published global Indigenous health literature. This has implications for generating investment into Indigenous health programs since the lack of such evidence limits our ability to assess the investment-case of interventions based on the criteria of cost-effectiveness, rather than solely for equity reasons or broader policy objectives. This is concerning given the significant disparities in health and access to health care that exist between Indigenous and non-Indigenous populations worldwide. Nonetheless, the evidence-base that does exist in the literature provides some isolated insights into the potential cost-effectiveness of specific types of interventions. There is potential for further work to both increase the use of economic evaluation in this area and methodological work to ensure that health economic methodologies are relevant to Indigenous populations.

### Limited Economic Research in the Field of Indigenous Health

A total of nineteen studies met the inclusion criteria for this review. Given the broad scope of the research question and search strategy, this depicts a very limited evidence-base from which to draw insights on the potential cost-effectiveness of interventions into Indigenous populations. This finding implies that investment into the area is largely being undertaken blind, based on assumptions rather than evidence of the cost and effectiveness of particular policies and interventions.

Three main reasons are offered here as potential factors explaining the lack of research in the field. First, there is a general lack of effectiveness studies in the field of Indigenous health, with the majority of research carried out in the field being observational rather than interventional in nature [35]. The findings of this review build upon previous literature that has emphasised the need to focus further research on finding effective interventions to improving the health of these unique groups. Second, the lack of studies could be a reflection on the attitudes of policy-makers and service providers in the area of Indigenous health that due to the severe inequalities faced by Indigenous population groups, interventions are justified purely on equity grounds rather than cost-effectiveness considerations. Such an argument overlooks the role that economic research could play in not only highlighting the relative importance of investing in Indigenous health, but providing guidance as to the best use of resources within the sector to maximise their impact. Third, as a result of the unique conceptions of health held by Indigenous populations, traditional economic evaluation methodologies may be inappropriate for the field of Indigenous health [8]. Specifically, it has been argued that traditional economic evaluation methods have failed to adequately accommodate the values, knowledge and beliefs of Indigenous populations such as those set out in the United Nations Declaration on the Rights of Indigenous People [8],[36]. Health benefits have generally been measured using individualistic and Westernised constructs of health, which have been shown, at least in the Australian context, to be distinctly different from the communitarian values of Indigenous culture [8]. This has potentially led to missed opportunities to improve indigenous health and wellbeing as the full range of benefits, costs and cost-savings that potentially arise from indigenous health interventions may not be captured. There is room for further refinement of economic methodologies to incorporate these concerns and particularly in the assessment of the applicability of existing health utility instruments, and potentially the development of new ones that may be more sensitive to Indigenous conceptions of health.

While the evidence-base identified in this review demonstrates that traditional health economic approaches can ostensibly be used to show the cost-effectiveness (or otherwise) of interventions aimed at improving Indigenous health, there has rarely been much attempt to incorporate Indigenous valuations of the potential gains from such programs.

Examples do exist in the literature of attempts to incorporate these values into economic evaluations of health interventions. The study of the Daruk-controlled midwifery service included above attempted to do so by taking a broader cost-consequence approach rather than restricting the analysis to narrowly defined health outcomes [11]. This involved a strong qualitative component to the study, which is an approach also taken by Jin et al. in their included study above [22]. Cost-consequences analyses or ‘the basket of goods approach’ has been viewed with some disdain in the health economic literature because of the potential for data mining and its inability to generate a clear decision rule [37]. It has been suggested however that this can be to some extent addressed by pre-specification of a conceptual framework in which the link between the multiple outcomes are linked to the intervention [38]. Further research into the suitability to of existing health economic techniques used to elicit preferences from target populations to Indigenous groups could provide a means to incorporate these values in a meaningful and rigorous way.

### Characteristics of Included Studies

While there was a limited amount of cost-effectiveness research of interventions to improve the health of Indigenous population groups, a number of conclusions can be drawn from the studies that were identified.

First, this review highlights that interventions into Indigenous populations, in particular rural and remote Indigenous communities, can be cost-effective, a broad but important finding given the often isolated and small populations of these groups when compared to non-Indigenous populations. Of the 27 interventions examined by included studies, 21 were deemed to be cost-effective or cost-saving while of the twelve interventions targeted solely to rural and remote populations, ten were found to be cost-effective or cost-saving by the respective studies. Being able to point to a body of evidence highlighting the cost-effectiveness of such interventions is important to justify widespread implementation of such programs on more than solely equity grounds and ensure that domestic debates on service provision are informed and based on the best available evidence.

The evidence-base drawn together by this review provides insights into particular interventions. Outreach programs were shown to be consistently cost-effective or cost-saving in all six interventions studied. These interventions were assessed relative to populations obtaining the services from alternative service providers. Thus they were found to be cost-saving or cost-effective despite often representing relatively high-costs for the health gains that occurred. They may be prohibitively costly in low-income environments outside of donor provision and it is also unclear how sustainable or community appropriate such models of service provision are. Explicit decisions need to be made by policy-makers in assessing the appropriateness of these services to local conditions.

The four injury prevention interventions were all found to be cost-effective as was the study of telehealth. Conversely, neither of two midwifery programs studied was found to be strictly cost-effective (one found non-significant cost-savings). Nor were the two studies focused solely on interventions in urban areas, highlighting the need for further work in this area. The majority of the interventions were targeted specifically at Indigenous groups (twenty-three interventions) and most were delivered through culturally specific medical providers, such as Aboriginal Medical Services in Australia and Canada. This is in line with findings from the literature that culturally specific services are more effective in reaching these populations [39].

### Limitations of this Review and included studies

While the strength of the review lies in the broad search strategy and research question, the heterogeneity of included studies limited the policy implications that could be drawn from the identified literature. The studies were of varied scope and included different notions of what constituted a cost-effective intervention. There are inherent difficulties in comparing the outcome of the studies when the notion of what constituted a cost-effective intervention varied so greatly between them.

The studies identified were largely drawn from high-income nations and focused at a primary-care level in rural or remote populations. There is a large gap in the literature for Indigenous populations of low and middle-income nations. Similarly, urban Indigenous communities have been largely overlooked in the literature despite these communities often representing the bulk of Indigenous populations within countries. In Australia, for example, it is estimated that the 60% of the ‘gap’ in health outcomes between the Indigenous and non-Indigenous populations is a result of the health of urban Indigenous communities [40]. The results of this review are consistent with previous studies that have pointed to a lack of studies in the area of urban Indigenous health relative to remote and rural communities [41]. Indigenous conceptions of health were rarely explicitly acknowledged in the cost-effectiveness literature. While the finding that the majority of included interventions were Indigenous-specific suggests that these factors may be implicitly be worked into most of the studies (at least to the extent that interventions are appropriately designed for these cultural groups), there is room to better include these ideas into general economic methodologies.

## Conclusion

Despite global commitments to reducing Indigenous health disadvantage, relatively little is known about what constitutes cost-effective investments into Indigenous populations around the world. Furthermore, the evidence that exists has often relied on pivotal evidence extrapolated from non-Indigenous settings and been based on methods that have not allowed for the values that such communities place on health to be included. Nevertheless, in light of the limited available evidence, this review suggests that interventions into these often hard to reach populations can be cost-effective. Further economic research has the potential to provide much needed guidance to policy-makers on resource allocation decisions and help improve the health of Indigenous people around the world but it needs to be based on the development of methods that incorporate values specific to the communities in question. .

## Supporting Information

Checklist S1 **PRISMA checklist.**
(DOC)
